# Supplementary material for: End Sequence Analysis Toolkit (ESAT) expands the extractable information from single-cell RNA-seq data
Source: Genome Res. 2016 Oct;26(10):1397–410. doi: 10.1101/gr.207902.116 (PMC5052061; doi:10.1101/gr.207902.116)
Supplement: Supplemental Material [file supp_gr.207902.116_Supp_Materials.tar.gz › ESAT-esat_v0.1/doc/html/dir_8530d4a580cb4d50cf384308e0640be3.html]

ESAT: java/broad/pda Directory Reference


ESAT


- Main Page
- Related Pages
- Packages
- Classes
- Files

All Classes Namespaces Files Functions Variables Enumerator Pages

- java
- broad
- pda

pda Directory Reference

Directory dependency graph for pda:

|  |  |
| --- | --- |
| Directories | |
| directory | annotation |
|  | |
| directory | countreads |
|  | |
| directory | datastructures |
|  | |
| directory | differentialExpression |
|  | |
| directory | enrichment |
|  | |
| directory | feature |
|  | |
| directory | gene |
|  | |
| directory | geneexpression |
|  | |
| directory | rap |
|  | |
| directory | rnai |
|  | |
| directory | samtools |
|  | |
| directory | seq |
|  | |
| directory | tools |
|  | |


---

Generated on Fri Aug 15 2014 15:11:16 for ESAT by  

 1.8.7
